# α-type-1 polarized dendritic cell-based vaccination in recurrent high-grade glioma: a phase I clinical trial

**DOI:** 10.1186/1471-2407-12-623

**Published:** 2012-12-27

**Authors:** Yasuto Akiyama, Chie Oshita, Akiko Kume, Akira Iizuka, Haruo Miyata, Masaru Komiyama, Tadashi Ashizawa, Mika Yagoto, Yoshiaki Abe, Koichi Mitsuya, Reiko Watanabe, Takashi Sugino, Ken Yamaguchi, Yoko Nakasu

**Affiliations:** 1 Immunotherapy Division, Shizuoka Cancer Center Research Institute, 1007 Shimonagakubo, Nagaizumi-cho, Sunto-gun, Shizuoka 411-8777, Japan; 2 Shizuoka Cancer Center Hospital, Division of Neurosurgery, Shimonagakubo, Nagaizumi-cho, Shizuoka, 411-8777, Japan; 3 Shizuoka Cancer Center Hospital, Division of Diagnostic Pathology, Shimonagakubo, Nagaizumi-cho, Shizuoka, 411-8777, Japan; 4 Division of blood stem cell transplantation, Shizuoka Cancer Center Hospital, Shimonagakubo, Nagaizumi-cho, Shizuoka, 411-8777, Japan

**Keywords:** Dendritic cell, Immunotherapy, High-grade glioma, HLA-A24, Phase I trial

## Abstract

**Background:**

High-grade gliomas including glioblastoma multiforme (GBM) are among the most malignant and aggressive of tumors, and have a very poor prognosis despite a temozolomide-based intensive treatment. Therefore, a novel therapeutic approach to controlling recurrence is needed. In the present study, we investigated the effect of activated dendritic cell (DC) (α-type-1 polarized DC)-based immunotherapy on high-grade glioma patients with the HLA-A2 or A24 genotype.

**Methods:**

Nine patients with recurrent high-grade gliomas including 7 with GBMs who fulfilled eligibility criteria were enrolled into a phase I study of monocyte-derived DC-based immunotherapy. HLA-genotyping revealed 1 case of HLA-A*0201 and 8 cases of A*2402. Enriched monocytes obtained using OptiPrep^TM^ from leukapheresis products on day1, were incubated with GM-CSF and IL-4 in a closed serum-free system, and activated on day6 with TNF-α, IL-1β, IFN-α, IFN-γ, and poly I/C. After pulsing with a cocktail of 5 synthetic peptides (WT-1, HER2, MAGE-A3, and MAGE-A1 or gp100) restricted to HLA-A2 or A24 and KLH, cells were cryopreserved until used. Thawed DCs were injected intradermally in the posterior neck at a dose per cohort of 1.0, 2.0 and 5.0× 10^7^/body.

**Results:**

The frequency of CD14^+^ monocytes increased to 44.6% from 11.9% after gradient centrifugation. After a 7-day-incubation with cytokines, the mean percentage of DCs rated as lin^-^HLA-DR^+^ in patients was 56.2 ± 19.1%. Most DCs expressed high levels of maturation markers, co-stimulatory molecules and type-1 phenotype (CD11c^+^HLA-DR^+^) with a DC1/2 ratio of 35.6. The amount of IL-12 produced from activated DCs was 1025 ± 443 pg/ml per 10^5^ cells. All 76 DC injections were well tolerated except for transient liver dysfunction with grade II. Six patients showed positive immunological responses to peptides in an ELISPOT assay, and positive skin tests to peptide-pulsed DC and KLH were recognized in 4 cases. The clinical response to DC injections was as follows :1 SD and 8 PD. Interestingly, the SD patient, given 24 DC injections, showed a long-term recurrence-free and immunological positive response period.

**Conclusions:**

These results indicate peptide cocktail-treated activated α-type-1 DC-based immunotherapy to be a potential therapeutic tool against recurrent high-grade glioma with mainly HLA-A*2402.

**Trial registration:**

Current non-randomized investigational trial UMIN-CTR UMIN ID: 000000914.

## Background

High-grade gliomas including glioblastoma multiforme (GBM) are among the most malignant and aggressive of tumors, and have a very poor prognosis and high recurrence rate with a mean survival time of less than 2 years even with the recent development of an intensive temozolomide (TMZ)-based treatment protocol [1,2]. Thus, a novel therapeutic approach to controlling recurrence and overcoming resistance to treatment is urgently needed for high-grade glioma patients.

Novel therapeutic approaches to controlling tumor recurrence by targeting crucial signaling molecules like TGF-β [3], JAK-STAT [4-6] and WINT [7], adhesion molecules like αβ integrin [8], and pro-angiogenic factors like VEGF and VEGFR [9], have been tried. However, a breakthrough has yet to be made. Meanwhile, immunotherapy is also recognized as a therapeutic tool against high-grade gliomas, and preliminary studies trying to suppress recurrence have been reported [10-13]. DC-based vaccines against high-grade gliomas have moderate effects in terms of a patient`s performance status (PS) or quality of life (QOL) [14], however the impact of such approaches is still not obvious because of a weak vaccine effect and poor PS.

Since the Provenge cancer vaccine against metastatic prostate cancer was approved by the FDA based on a its impact on overall survival in a phase III randomized study, the life-prolonging effect of DC vaccines without significant side effects has been receiving attention [15,16]. Additionally, an important point regarding a successful DC-based cancer vaccine is still the full maturation of DCs because it affects significantly the function of DCs and anti-tumor effect directly. However, a precise evaluation for maturation has not established and therefore various factors are utilized as so-call maturation markers.

In the present study, we have manufactured α-type-1 polarized DCs, activated by a combination of maturation reagents like TNF-α, IL-1β, IFN-α, IFN-γ and polyI/C. Αlpha-type-1 polarized DCs were first reported by Mailliard et al., who found a large amount of IL-12 in supernatant [17]. Additionally, Okada et al. reported that an α-type-1 polarized DC vaccine treated with HLA-A2 peptides was useful for controlling relapse in cases of high-grade glioma [18]. Based on these results, we considered that an α-type-1 DC vaccine also could have a therapeutic effect on HLA-A24^+^ glioma patients, and performed a phase I clinical trial of HLA-A24-restricted peptide cocktail-pulsed DC-based immunotherapy against high-grade glioma. Here we describe the safety and efficacy of an α-type-1 DC-based vaccine against recurrent high-grade glioma.

## Methods

### Patient characteristics and eligibility criteria

Nine patients with recurrent high- grade glioma were enrolled in a phase I clinical trial of a peptide cocktail-pulsed α-type1-polarized DC-based vaccine approved by the Institutional Review Board of the Shizuoka Cancer Center, Shizuoka, Japan. All patients gave written informed consent. Patients` characteristics are listed in Table 1. Inclusion criteria were: i) histological diagnosis of high-grade glioma (WHO grade III or IV) [19], ii) poor response to standard treatment [surgical therapy (ST) + radiation therapy (RT) + temozolomide], iii) 20 y.o. ≦ age < 80 y.o., iv) performance status ≦ 2, v) evaluable lesions in imaging diagnosis, vi) HLA genotyping ; HLA-A2 or A24, vii) no severe systemic organ dysfunction, viii) no severe hematostatic dysfunction, ix) informed consent obtained from patients, and x) life expectancy > 6 months. Minimum doses of corticosteroid (dexamethasone up to 1 mg/day) were permitted for patients with neurological deficits due to mass effects by the lesions.

**Table 1:** Phase I study of DC-based therapy against high-grade glioma

**Patient no.**	**Age**	**Sex**	**Previous therapy**	**PS**	**Pathological diagnosis (WHO grade)**
**1**	**69**	**M**	**ST, CT**	**1**	**GBM (IV)**
**2**	**49**	**M**	**ST, CT, RT**	**0**	**AO (III)**
**3**	**38**	**M**	**ST, CT, RT**	**2**	**GBM (IV)**
**4**	**32**	**F**	**CT, RT,**	**2**	**AA (III)**
**5**	**61**	**M**	**ST, CT, RT**	**1**	**GBM (IV)**
**6**	**32**	**M**	**ST, CT, RT**	**1**	**GBM (IV)**
**7**	**73**	**M**	**ST, CT, RT**	**1**	**GBM (IV)**
**8**	**53**	**F**	**ST, CT,**	**1**	**GBM (IV)**
**9**	**31**	**M**	**ST, CT,**	**1**	**GBM (IV)**

ST; surgical therapy, CT; chemotherapy, RT; radiation therapy.

GBM; glioblastoma multiforme, AO; anaplastic oligodendroglioma.

AA; anaplastic astrocytoma.

Exclusion criteria were : i) severe systemic infection and organ failure, ii) pregnancy, iii) severe immunological disorders (autoimmune disease, immunosuppression), iv) multiple cancers, and v) anaphylaxis to synthetic peptides. All the patients received intradermally (i.d.) 4 DC vaccines at the posterior neck weekly, and toxicity was checked. DCs were injected in a dose-escalation design at a dose level per cohort of 1.0, 2.0, and 5.0 ×10^7^/body/shot. The injected DC number was calculated from the percentage of Lin^-^HLA-DR^+^ gated populations in a FACS analysis.

### Preparation of DCs and peptides

The procedures for preparing the DC vaccine were performed within a high efficiency particle arrestance (HEPA)-filter clean-air barriered good manufacturing practice (GMP) cell processing facility. A standard operation procedure (SOP) for DC vaccine production was established according to institutional GMP-based guidelines. The procedures have been described before [20]. Briefly, leukapheresis products were washed and centrifuged using density-adjusted OptiPrep™ (Axis-Shield PoC, Oslo, Norway), and the monocyte layer at the top was retrieved. On day1, cells were transferred to an X-fold culture bag (Nexell, Irvine, CA) and cultured in the presence of GM-CSF at 50 ng/ml (CellGenix, Freiburg, Germany) and IL-4 at 50 ng/ml (CellGenix) in X-VIVO15 serum-free medium (Biowhittaker, Walkersville, MD). On day6, cells were activated by the addition of TNF-α at 10 ng/ml (CellGenix), IL-1β at 10 ng/ml (CellGenix), IFN-α at 3000 U/ml (Dainippon Sumitomo Pharma Co. Ltd, Osaka, Japan), IFN-γ at 1000 U/ml (Shionogi & Co. Ltd, Osaka, Japan), and poly I/C at 20 μg/ml (Amersham Biosciences Corp., Piscataway, NJ). On day8, harvested cells were pulsed with a cocktail of 5 synthetic peptides (25 μg/ml each) restricted to HLA A2 or A24 and KLH (25 μg/ml, Intracell, Frederick, MD). Finally, DC-enriched cells were washed and cryopreserved in Cryocyte bags (Baxter Healthcare Co., Deerfield, IL) until used. The purity of CD14^+^ cells was evaluated with a flow cytometer (FACSCalibur, Becton-Dickinson Co., CA) before and after OptiPrep™ separation. The percentage of DCs was rated as the lin^-^HLA-DR^+^ population (lineage antibodies including CD3, CD14, CD16, CD19, CD20, CD56 ; BD Biosciences, San Jose, CA). The frequencies of the DC-related markers were determined using various antibodies for CD1a, CD11c, CD33, CD40, CD54, CD80, CD86, CD123, CD205, CD207, CMRF44, CMRF56, E-cadherin CCR7, HLA-class I, and HLA-DR (BD Biosciences). The ratio of DC1 (CD11c^+^HLA-DR^+^) and DC2 (CD123^+^HLA-DR^+^) was calculated using a flow cytometric analysis reported by Ferrari et al. [21].

The following peptides restricted to HLA-A2 or A24 were synthesized according to GMP standards by Multiple Peptide Systems, CA: HLA-A2: WT1126-134 (RMFPNAPYL), WT1235-243 (CMTWNQMNL), gp100209-217 (IMDQVPFSV), HER2369-377 (KIFGSLAFL), MAGE-A3271-279 (FLWGPRALV) ; HLA-A24: WT1235-243M (CYTWNQMNL), WT1235-243 (CMTWNQMNL), HER263-71 (TYLPTNASL), MAGE-A1135-143 (NYKHCFPEI), MAGE-A3195-203 (IMPKAGLLI).

### IL-12p70 production assay

Cultured mature DCs were collected, and plated in a round-bottomed 96-well microplate at 5 × 10^4^ cells/well. To stimulate IL-12p70 production by DCs, a CD40 ligand-expressing mouse plasmacytoma cell line, J558, was added at 1 × 10^5^/well. CD40L-transfected J558 cells were kindly supplied by Dr. Kalinsky, the University of Pittsburgh Cancer Institute, Pittsburgh, PA. Both cells were incubated for 24 hrs. Finally, supernatants were collected and IL-12p70 levels were measured using an ELISA kit specific for human IL-12p70 (Endogen, Woburn, MA).

### Tumor antigens and other antigens in tumor tissues prior to vaccination

High-grade glioma tissues were obtained from patients who gave written informed consent. The expression of tumor antigens including MAGE-A1, A3, WT-1, HER2 and gp100 was investigated using a non-quantitative RT-PCR and an immunohistochemical (IHC) analysis as described previously [22]. The primer sequences used were 5’-CGGCCGAAGGAACCTGACCCAG-3’ and 5’-GCTGGAACCCTCACTGGGTTGCC-3’ for MAGEA-1, 5’-TGGAGGACCAGAGGCCCCC-3’ and 5’-GGACGATTATCAGGAGGCCTGC-3’ for MAGE-A3, 5’-TCTTCAGAGGCATTCAGGATGTGC-3’ and 5’-GAGTCCTGGTGTGGGTCTTCAGGT-3’ for WT-1, 5’-CTATGGCTGCCTCTTAGACCATGTCC-3’ and 5’-AAAGTCATCAGCTCCCACACAGTCAC-3’ for HER2, 5’-TAGTGGAACCCTGATCTCTC-3’ and 5’-TCAGGGATAGGTAGCTCTCT-3’ for gp100, and 5’-GGCTACAGCTTCACCACCAC-3’ and 5’-GTACTTGCGCTCAGGAGGAG-3’ for beta-actin. HLA-class I protein expression was also evaluated using an IHC analysis. Antibodies against MAGE-A1 (Thermo scientific, Flemont, CA), MAGE-A3 (Abnova, Taipei, Taiwan), WT-1 (DakoCytomation, Glostrup, Denmark), HER1 (DakoCytomation), gp100 ((DakoCytomation) and human HLA class I (Hokudo Co. Ltd., Sapporo, Japan) were used as the primary antibody and a goat anti-mouse or anti-rabbit IgG antibody, as the secondary antibody. Horseradish peroxidase (HRP) and hydrogen peroxide were utilized for color development according to the manufacturer`s instructions (Vectastatin kit, Vector Lab., CA).

### Clinical and immunological monitoring

Clinical assessment was performed using RECIST criteria [23] as follows; complete response (CR) : disappearance of lesions at 4 wks, partial response (PR): 30% decrease in sums of longest diameters at 4 wks, stable disease (SD): neither PR nor PD criteria met, progressive disease (PD): 20% increase in sums of longest diameters. Clinical response was rated as maximal through the DC vaccinations. The patients received up to 10 injections on the condition that at least one measurable lesion showed more than a SD response and/or an ELISPOT assay performed after 4 injections indicated a positive response for more than 1 peptide. Adverse effects were evaluated according to the NCI Common toxicity criteria after 4 DC injections. Peripheral blood mononuclear cell (PBMC) samples were harvested before and 29, 78, 134 and 190 days after the start of DC injections for immunological monitoring. All patients were followed regularly throughout, and an MRI was performed every 2 to 3 months during the vaccination period.

### ELISPOT assay

The ELISPOT assay was performed using PBMCs drawn prior to vaccination and after 4 DC injections. Briefly, PBMCs were incubated in a 24-well culture plate and divided into non-adherent and adherent cells. Adherent cells were treated with a peptide cocktail and β2-microglobulin, and co-cultured with non-adherent cells in the presence of IL-2 and IL-7. On day7, non-adherent cells were re-stimulated with peptide-pulsed adherent cells. On day14, responder cells (5×10^4^/well) were stimulated with HLA-A2 or A24 peptides in a 96-well culture plate coated with anti-IFN-γ antibody (MABTECH AB, Nacka, Sweden) overnight. Finally, positive spots stained with anti-IFN-γ antibody were measured using the KS ELISPOT system (Carl Zeiss AG, Overkochen, Germany). A HLA-A2- (GILGFVFTL) or A24-restricted CMVpp65 peptide (RYSIFFDY) was used as a positive control. The spot number per well of peptide-stimulated CTLs was compared to that of a negative well without peptide using Student’s paired two-tailed *t-*test.

### Intracellular cytokine staining

PBMCs were stimulated with 25 ng/ml of PMA (Sigma) and 1 μg/ml of ionomycin (Sigma) for 5hrs in a 96-well culture plate. After the stimulation, cells were stained with FITC-anti-CD4 MoAb, and subsequently intracellular staining was performed with fix/permealization buffer and PE-labeled anti-IFN-γ or anti-IL-4 MoAb (Pharmingen,San Diego, CA). Finally, the ratio of Th1 (IFN-γ^+^) and Th2 (IL-4^+^) was calculated in PBMC samples obtained from patients.

### DTH reactions

The HLA-A2 or A24 peptide solution and KLH at a dose of 50 μg/ml were injected intradermally into the forearm and the redness and induration at the injection site were measured on days 29, 78, 134 and 190 after the 1st DC injection. 1 × 10^6^ DCs treated with peptides were added to DTH antigens after the start of the vaccination. PPD was used as a positive control.

### Statistical analysis

Statistical differences were analyzed using Student’s paired two-tailed *t-*test. Values of *p* < 0.05 were considered significant.

## Results

**Patient characteristics** The nine patients consisted of 7 males and 2 females, and mean age was 48.6 ± 16.4. Eight cases were HLA-A*2402 in genotype. Previous therapy including ST, RT and chemotherapy (CT) was performed in all patients. Histologically, there were 6 GBMs (WHO grade IV), 1 anaplastic astrocytoma (AA) (grade III) and 1 anaplastic oligodendroglioma (AO) (grade III) (Table 1).

### Characterization of tumor antigen expression

**Figure 1:**
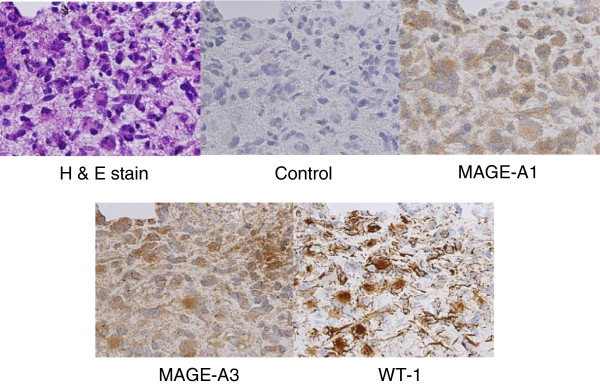
**Immunohistochemical analysis of tumor antigens in resected tumor tissue from patient 6.** Many positively stained pleomorphic giant cells and their processes were shown. Each primary antibody against MAGE-A1, MAGE-A3, and WT-1 was used at a dilution of 1:10 ~ 1:100. Mouse IgG antibody was used as a control. H & E stain; hematoxylin and eosin stain. Magnification ×400.

An analysis of tumor antigen expression by RT-PCR and IHC was performed in 6 evaluable cases. The antigen expression was determined as positive, when either the RT-PCR or IHC analysis was positive. All 5 tumor antigens including MAGE-A1, -A3, HER2, gp100 and WT1 were positive in 5 cases except for patient 5 in which 3 antigens were identified in the tumor (Table 2). A representative case of tumor expression analyzed by IHC, patient 6, showed strong reactions to MAGE-A1, MAGE-A3, and WT-1 antigens (Figure 1). The HLA-class I protein was positive in all cases, but the expression level was different (data not shown).

**Table 2:** Tumor antigen and HLA-class I expression in resected tumors

**Patient**	**hHLA-typing**	**Tumor antigen expression**	**HLA-class I**^**a**^
**1**	A*2402	**N. D.**	**N. D.**^**b**^
**2**	A*2402	**5/5 (M1, M3, HER2, gp100, WT-1)**	**++**
**3**	A*2402	**5/5**	**+**
**4**	A*2402	**N. D.**	**N. D.**
**5**	A*2402	**3/5 (M3, HER2, WT-1)**	**+**
**6**	A*2402	**5/5**	**+**
**7**	A*0201	**5/5**	**+**
**8**	A*2402	**N. D.**	**N. D.**
**9**	A*2402	**5/5**	**+**

^**a**^HLA-class I expression was checked on IHC, ^b^ N. D. ; not done.

### DC production efficiency and characterization

**Figure 2:**
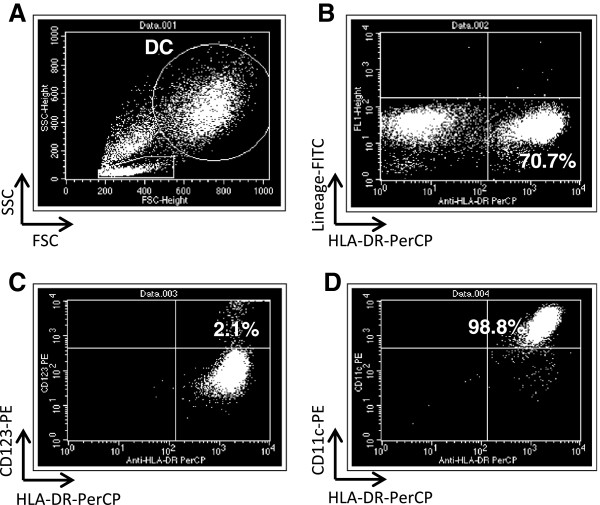
**Characterization of cultured α-type-1 DCs from patient 6 by flow cytometry.** The mean percentage of DCs rated as lin^-^HLA-DR^+^ is shown in **B**. **A**; dot gram of whole cultured cells. The DC gate is shown as a large and widely distributed population. **B**; DC population determined as lin^-^HLA-DR^+^ cells. **C**; DC2 (CD123^+^HLA-DR^+^) and **D**; DC1 (CD11c^+^HLA-DR^+^) is shown, respectively.

Through gradient-centrifugation using Optiprep, the frequency of CD14^+^ cells improved from 11.9% to 44.6%, an increase of 4 fold (Table 3). The mean percentage of DCs rated as lin^-^HLA-DR^+^ (Figure 2) and the DC1/DC2 ratio in glioma patients was 56.9 ± 19.1% and 35.6, respectively, comparable to a previous phase I DC vaccine study for metastatic melanomas. Comparing obtained DC numbers to starting total cell numbers, the recovery rate was determined as 8.79%, also comparable to the previous study. Importantly, IL-12p70 levels produced by DCs through CD40 ligand stimulation were remarkably high, more than 1,000 pg/ml, which indicated a fully mature phenotype of the obtained DCs (Table 3). The frequencies of the DC-related markers were determined in gated lin^-^HLA-DR^+^ cells as follows; CD1a 16.3 ± 13.2%, CD40 95.9 ± 5.1%, CD54 99.3 ± 1.2% , CD80 92.4 ± 15.5%, CD83 44.7 ± 19.5%, CD86 99.6 ± 0.3%, CD205 73.9 ± 12.1%, CD207 92.3 ± 11.6%, HLA-class I 99.0 ± 1.2%, which also demonstrated the fully mature phenotype (Table 4).

**Table 3:** Efficiency of α-type-1 DC production procedures

**Case numbers**	**Starting total cell no. (×10**^**9**^**)**	**Optiprep (CD14**^**+**^**%)**		DC rate (%)* (lin^**-**^**HLA-DR**^**+**^**)**	**Total DC numbers (×10**^**8**^**)**	**Recovery rate (%)**	**DC1/DC2 ratio**	**Il-12p70 level (pg/ml)**
		**pre**	**post**					
**9**	**6.50±3.4**	**11.9±9.4**	**44.6±21.8**	**56.1±19.0**	**4.22±1.7**	**8.79±5.1**	**35.6±39.6**	**1025±443**

* The frequency of DCs was determined from lineage (CD3, CD14, CD16, CD19, CD20, CD56)^-^HLA-DR^+^ cell population.

**Table 4:** **Phenotypical analysis of α-type-1 DCs after a 7-day culture**

**Markers**	Frequency (%)*	**Markers**	**Frequency (%)**	**Markers**	**Frequency (%)**
**CD1a**	**16.3±13.2**	**CD80**	**92.4±15.5**	**CMRF44**	**3.7±2.4**
**CD11c**	**99.3±0.6**	**CD83**	**44.7±19.5**	**CMRF56**	**37.1±11.2**
**CD33**	**7.0±3.8**	**CD86**	**99.6±0.3**	**E-cadherin**	**13.1±7.4**
**CD40**	**95.9±5.1**	**CD205**	**73.9±12.1**	**CCR7**	**6.0±1.9**
**CD54**	**99.3±1.2**	**CD207**	**92.3±11.6**	**HLA-class I**	**99.0±1.2**

* Each value represents the frequency of marker-positive cells in lineage^-^HLA-DR^+^ gated population.

### ELISPOT assay

**Figure 3:**
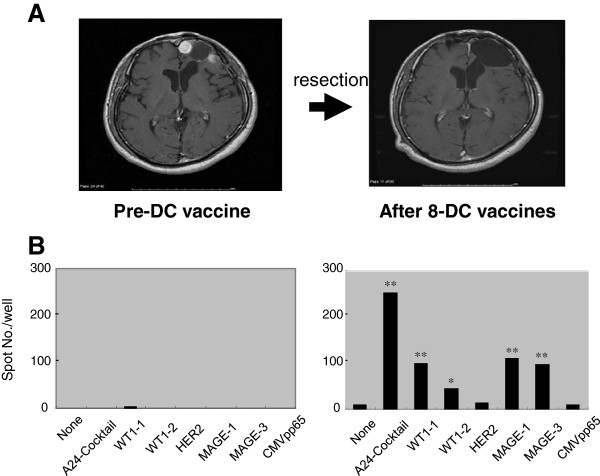
**Effect of DC vaccines on tumor progression and CTL induction in patient 2. A**; Maintenance of progression-free status for as long as 34 months by DC vaccines after surgical resection as revealed by MRI. **B**; Remarkable CTL activity after 8 DC vaccines as revealed by ELISPOT assay. The spot number per well of peptide-stimulated CTLs was compared to that of a negative well without peptide using Student’s paired two-tailed *t-*test. Statistically significant compared to no peptide, *; *P* < 0.05, **; *P* < 0.01. None; no peptide.

CTL precursors against synthetic peptides were recognized after the vaccination in 6 evaluable cases (Table 5). Notably, 3 cases demonstrated CTL responses to more than 3 peptides, and patient 2 showed a durable SD without relapse for 34 months after the start of the vaccination, with a remarkably high CTL response to 4 HLA-A24 peptides (Figure 3).

**Table 5:** Immunological monitoring in high-grade glioma patients

**Patient no.**	**DC injection (times)**	**Side effect**	**ELISPOT**	**DTH**			**Th1/Th2 balance**^**a**^	**Response**
				**Peptide**	**DC**	**KLH**		
**1**	**1×10**^**7**^**(12)**		**1/5 (MAGE-1)**		**+**	**+**	**9.38**	**PD**
**2**	**1×10**^**7**^**(24)**		**4/5 (WT1-1, 1–2, MAGE-1, 3)**	**++**	**++**	**+++**	**0.3**	**SD**
**3**	**1×10**^**7**^**(4)**		**N. D.**^**b**^				**9.67**	**PD**
**4**	**2×10**^**7**^**(5)**	**Hepatic (II)**	**3/5 (WT1-1, 1–2, MAGE-3)**				**4.09**	**PD**
**5**	**2×10**^**7**^**(6)**		**0**		**+**		**6.07**	**PD**
**6**	**2×10**^**7**^**(4)**		**0**				**2.77**	**PD**
**7**	**5×10**^**7**^**(7)**		**1/5 (gp100)**			**+**	**7.08**	**PD**
**8**	**5×10**^**7**^**(6)**		**3/5 (WT1-2, HER2, MAGE-3)**				**71.4**	**PD**
**9**	**5×10**^**7**^**(6)**		**2/5 (MAGE-1, 3)**	**++**	**+**	**+**	**7.55**	**PD**

^**a**^ The value shows Th1/Th2 ratio prior to DC vaccination. ^b^ N. D. ; not done. PD ; progressive disease, SD ; stable disease.

### Th1 and Th2 balance after DC vaccination

In all 9 cases, the balance of Th1 and Th2 shifted more to Th1 (mean ratio; 13.1 ± 7.3) before DC injections (Table 5). Unfortunately, PBMC samples were not obtained after the vaccinations in 8 cases. No correlation of Th1/Th2 ratio to ELISPOT responses was observed.

### DTH

**Figure 4:**
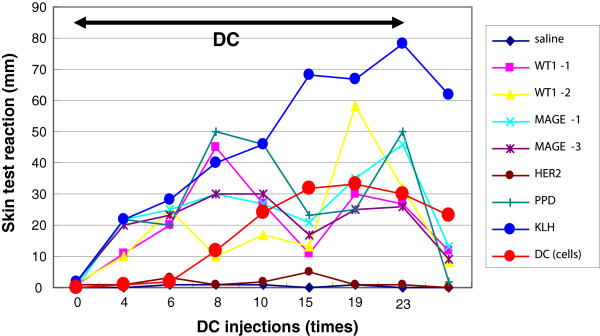
**Long-term activation of DTH to peptides and KLH through the course of DC vaccinations in patient 2.** The responses to each peptide, KLH, and DCs increased to a plateau, and responses to KLH and DCs remained highly positive even more than 2 years after the start of the vaccination, despite that the response to the peptides went down after the vaccinations ceased.

Positive DTH tests were verified in 4 patients against all peptides, 4 against DCs treated with peptides, and 4 against KLH (Table 5). Specifically, 2 patients (2 and 9) tested positive for all 3 antigens, and patient 2 exhibited very strong reactions. Surprisingly in patient 2, after the start of the vaccination, the responses to each peptide, KLH and DCs increased to a plateau, and responses to KLH and DCs remained highly positive even after more than 2 years, despite that the response to peptides went down after the vaccination ceased (Figure 4).

### Adverse effects of the DC vaccine

Safety was assessed after 4 DC injections in all 9 cases. Mild hepatic dysfunction (grade II) was seen in 1 case, however it was only temporary and disappeared in spite of the continuance of DC injections. No clinical symptoms of autoimmune disease were found (Table 5).

### Clinical response

Clinical response was rated as maximal throughout the DC vaccinations. Seven of 8 showed PD due to a rapid progression of the disease (Table 5). Patient 2 demonstrated a long SD after total resection for more than 2 years, in which strong CTL and DTH responses were recognized throughout the vaccination period.

## Discussion

Since sipuleucel-T (Provenge, Dendreon), an autologous cellular immunotherapy, was approved by the U.S. Food and Drug Administration (FDA), the significant effect of DC-based vaccines on overall survival in metastatic castration-resistant prostate cancer patients has attracted much attention despite a low rate of clinical response [24,25].

In early phase trials, sipuleucel-T showed good safety, but a weak anti-tumor response which was not impressive compared with chemotherapeutic regimens. However, the last double-blind, placebo-controlled, multicenter phase III trial of the sipuleucel-T vaccine clearly demonstrated a significant survival benefit for metastatic prostate cancer. Based on these observations, the major focus of cancer-specific DC-based immunotherapy has been shifting toward the improvement of overall survival and QOL including performance status.

With regard to the development of DC-based immunotherapy, many studies have been performed on relapsed or advanced high-grade gliomas since 2000 [26-29]. In most studies, monocyte-derived DCs treated with autologous tumor lysate have been utilized, and a beneficial effect on overall survival to some degree was obtained, but the objective response rate was still low. Reasons for the use of tumor lysate include; i) poor identification of high-grade glioma-specific tumor antigens, ii) independence of tumor lysate from HLA-restriction, and iii) practicality in terms of personalized medicine. However, disadvantages include the absence of an evaluable target antigen and immunological monitoring methods, and no validated protocol for lysate production from tumor tissues. Taking these issues into consideration, immunogenic synthetic peptides still seem to have an advantage over tumor lysate.

DC maturation status is becoming an important issue, and IL-12p70-producing ability would be a key factor to a successful DC vaccine for high-grade glioma [30-33]. Mailliard et al. reported that very efficient IL-12-producing DCs, so-call α-type-1 polarizing DCs, can be obtained by combining cytokines like IL-1β, TNF-α, IFN-α, IFN-γ and polyI/C with GM-CSF and IL-4, and the DCs induced remarkably stronger CTLs than conventional methods [17]. Based on these observations, Okada et al. [18] applied α-type-1 polarizing DCs to a phase I/II clinical trial for 22 cases of HLA-A2^+^ high-grade glioma, where HLA-A2 peptides like EphA2, Il-13R-a2, YKL-40 and gp100 were used, and obtained 2 objective responders and 9 cases with progression-free survival for at least 12 months. These results demonstrated that peptide-cocktail-based α-type-1 polarizing DCs were a better therapeutic tool than tumor-lysate-based DCs. More importantly, IL-12 production by α-type-1 polarizing DCs was shown to be positively correlated with the time to progression, which indicated IL-12-producing ability to be a potential prognostic factor.

In the present study, we also utilized α-type-1 polarizing DCs for HLA-A24^+^ recurrent high-grade glioma patients in a phase I trial. DC production was performed in a clean-air barriered good manufacturing practice (GMP) cell processing facility, and under standard operation procedures (SOP) for DC production according to institutional GMP-based guidelines. First, as to DC production efficiency, the mean number of obtained DCs per patient and mean DC yield was 4.22 x10^8^ and 8.79%, respectively which was comparable to the yield (3.4%) reported by Szmania et al. [34].

Previously, we reported a phase II trial of a DC vaccine against HLA-A24^+^ metastatic melanoma, and demonstrated that the number of DC injections was significantly associated with the prognosis [35]. This result suggests the total dose of qualified DCs to be a key factor to a successful vaccination. The longer patients are given DC vaccines, the better their prognosis will be. Therefore, the yield of DCs, namely the recovery rate from a leukapheresis product, is important. Second, IL-12 production levels were 1025 ± 443 pg per 10^5^ DCs, higher than the levels (< 500 pg) reported by Okada et al. [18]. A total of 74 DC injections were well-tolerated without significant adverse effects, and finally, safety and feasibility were verified. We also found a SD case with a relapse-free period of more than 2 years after tumor resection, which interestingly showed long positive DTH reactions against peptides, KLH and even DCs treated with peptides.

Recent progress in the clinical trial of DC vaccines against malignant glioma should be addressed. First, as Ardon et al. reported, the integration of autologous DC-based immunotherapy into the treatment of newly diagnosed GBM patients is ongoing [11]. The efficacy of DC vaccines in combination with RT and CT might be potentiated in such a study. Second, the potential of DC vaccines in combination with chemotherapy has been stressed. Wheeler et al. [36] demonstrated a strong link between the predominant T-cell effectors and chemosensitivity in GBM tumors. In the near future, to improve the vaccine-induced benefits and relapse-free period, optimal combinations of DC vaccines and chemotherapy need to be developed.

Finally, given the success of sipuleucel-T trials, multi-centered phase III randomized studies of specific peptide- or tumor lysate-treated DCs against high-grade glioma are strongly recommended.

## Conclusions

In the present study, we investigated the effect of immunotherapy based on α-type-1 polarizing DCs on high-grade glioma patients with mainly HLA-A*2402. Nine patients with high-grade glioma (1 case of HLA-A*0201, 8 of A*2402) were enrolled into a phase I study and given HLA-A2 or A24-peptide cocktail-pulsed mature DCs, which produced a large amount of IL-12. Positive immunological responses to peptides in an ELISPOT assay were found in 6 cases, and positive skin tests to peptide-pulsed DC and KLH were recognized in 4 cases. The clinical response to DC injections was 1SD and 8 PD. All 76 DC injections were safely administered to patients. These results suggested that peptide cocktail-treated α-type-1 DC-based immunotherapy was feasible, and had potential as a therapeutic tool against HLA-A24^+^ high-grade glioma.

## Abbreviations

DC: Dendritic cell; HLA: Human leukocyte antigen; FDA: Food and drug administration; IRB: Institutional review board; GM-CSF: Granulocyte macrophage-colony-stimulating factor; IL: Interleukin; KLH: Keyhole limpet hemocyanin; IHC: Immunohistochemistry; CTL: Cytotoxic T cell; DTH: Delayed-type hypersensitivity; CR: Complete remission; PR: Partial remission; SD: Stable disease; PD: Progressive disease; ELISA: Enzyme-linked immunosorbent assay; IFN: Interferon; HRP: Horseradish peroxidase; PS: Performance status; PBMC: Peripheral blood mononuclear cell.

## Competing interests

The authors have no competing interests to declare.

## Authors’ contributions

YA participated in the design of the study and drafting of the manuscript and was responsible for completing the study. CO, AK, HM, and YA carried out the apheresis and cell processing and were responsible for DC production. KM and YN were responsible for the clinical side of the study. CO, AI, HM, YM, and TA participated in the design of the experiments and performed the biological assays. TS and RW contributed to the pathological diagnosis. YN and KY reviewed the manuscript. All authors read and approved the final draft.

## Pre-publication history

The pre-publication history for this paper can be accessed here:

http://www.biomedcentral.com/1471-2407/12/623/prepub
